# Interfacial Engineering of Pickering Emulsion Co-Stabilized by Zein Nanoparticles and Tween 20: Effects of the Particle Size on the Interfacial Concentration of Gallic Acid and the Oxidative Stability

**DOI:** 10.3390/nano10061068

**Published:** 2020-05-30

**Authors:** Zijun Zhao, Wenbo Wang, Jie Xiao, Yunjiao Chen, Yong Cao

**Affiliations:** 1 Guangdong Provincial Key Laboratory of Nutraceuticals and Functional Foods, College of Food Science, South China Agricultural University, Guangzhou 510642, China; philoacademic@scau.edu.cn (Z.Z.); yunjiaochen@scau.edu.cn (Y.C.); caoyong2181@scau.edu.cn (Y.C.); 2 Guangdong Laboratory for Lingnan Modern Agriculture, Guangzhou 510642, Guangdong, China; 3 College of Electronic Engineering, South China Agricultural University, Guangzhou 510642, China; wwb@scau.edu.cn

**Keywords:** oxidative stability, Pickering emulsion, interfacial concentration of phenolic antioxidant, zein nanoparticles, particle size

## Abstract

Lipid oxidation is still one of the major food-safety issues associated with the emulsion-based food systems. Engineering the interfacial region is an effective way to improve the oxidative stability of emulsion. Herein, a novel Pickering emulsion with strong oxidative stability was prepared by using zein nanoparticles and Tween 20 as stabilizers (ZPE). The modulation effects of the particle size on the distribution of gallic acid (GA) and the oxidative stability of ZPE were investigated. In the absence of GA, Pickering emulsions stabilized with different sizes of zein nanoparticles showed similar oxidative stability, and the physical barrier effect took the dominant role in retarding lipid oxidation. Moreover, in the presence of GA, ZPE stabilized by zein nanoparticles with the averaged particle size of 130 nm performed stronger oxidation than those stabilized by zein nanoparticles of 70 and 220 nm. Our study revealed that the interfacial concentration of GA (GA_I_) was tuned by zein nanoparticles due to the interaction between them, but the difference in the binding affinity between GA and zein nanoparticles was not the dominant factor regulating the (GA_I_). It was the interfacial content of zein nanoparticles (Γ), which was affected by the particle size, modulated the (GA_I_) and further dominated the oxidative stability of ZPEs. The present study suggested that the potential of thickening the interfacial layer to prevent lipid oxidation was limited, increasing the interfacial concentration of antioxidant by interfacial engineering offered a more efficient alternative.

## 1. Introduction

Lipid oxidation is a common deterioration reaction occurring in emulsion-based food systems [1]. Consisting of small lipid droplets dispersed in an aqueous phase, oil in water emulsions exposes a much larger surface area to the surrounding aqueous phase, as compared to the blank oil. As unsaturated lipids and aqueous pro-oxidants, such as metal ions, come into close contact at the interface region, it has been widely accepted that the oil–water interface is where the lipid oxidation initiates and propagates, and the physicochemical properties of the interfacial layer is thus extremely relevant to lipid oxidation [1,2,3,4]. Researchers have therefore focused on manipulating interfacial properties to improve the oxidative stability of emulsions [5,6].

Some studies have indicated that the thickness of the interface layer actively impacted the lipid oxidation stability of emulsions [5,7]. Thick interfacial layer inhibited the diffusion of hydrophilic pro-oxidants (such as transition metals) to unsaturated lipids within emulsion droplets, thereby retarding the lipid oxidation. The thickness of the interfacial layer also affected the mass transfer and location of other molecular species involved in lipid oxidation reactions, such as lipid hydroperoxides, free radicals, and antioxidants, thereby interfering with the oxidation reaction [2,8]. In surfactants-based emulsions, small-molecular-weight emulsifiers decrease the interfacial tension and tend to adsorb and diffuse at the oil–water interface instantaneously, and the adsorption/desorption process is dynamic within a very short time. Surfactant-based macro-emulsions, nano-emulsions, and microemulsions show the emulsion droplet sizes ranging from 0.5 to 50 μm, 10 to 500 nm, and 1~200 nm, respectively [9]. Experiments have shown that the rate of lipid oxidation in surfactant stabilized oil-in-water emulsions can be decreased by increasing either the head-group or tail-group dimensions of the adsorbed surfactants, which was attributed to their ability to form a thicker interfacial layer that inhibited interactions between pro-oxidants and lipids [4]. It has also been suggested that thicker interfacial protein layers (caseinate) inhibited lipid oxidation more effectively than those thinner layers (whey or soy protein) [1]. Consequently, developing emulsions with stronger lipid oxidation stability via forming thicker interfacial layer was proposed.

Surface-active particles with suitable wettability can replace traditional emulsifiers as emulsion droplet stabilizers, and emulsion systems stabilized by solid particles are termed as Pickering emulsions. The formation of Pickering emulsions does not need surfactants, while the surfactant could be added into the Pickering emulsion for better physical stability. Actually, Pickering emulsions co-stabilized with particles and surfactants have been emerging as a research hotspot and widely accepted in the research field [10,11]. Such systems are still defined as Pickering emulsions due to the fact that the properties of this emulsion system are more like Pickering emulsion in terms of droplet size, polydispersity, and especially the interfacial properties. In such systems, the coexistence of surfactants facilitated the loading efficiency of particles stabilized ones by means of changing the wettability of particles to a more suitable value, decreasing the interfacial tension at the early stage of the emulsification process, facilitating the migration of particles onto the interface, etc. With an appropriate mass ratio of nanoparticles and surfactants, the physical and chemical stability of the emulsion could be improved [10,11].

Unlike surfactants, the solid particles (with the particle size ranging from 10 nm to 100 μm) adsorbed at the oil–water interface irreversibly, and the emulsion droplet size ranged from 10 to 1000 μm [6]. A major advantage of the Pickering emulsion over the surfactant stabilized one lies in its high resistance against coalescence [6]. As the thickness of interfacial layer depends on the size of solid particles, Pickering emulsion is gaining great interest in recent studies for its thicker interfacial layers (ranging from 10 nm to 100 μm) than surfactants-based emulsions (1~50 nm) [5,12,13,14,15,16]. However, mounting evidences have suggested that the oxidation stability was not always relevant to the thickness of interfacial layer. Berton-Carabin et al. confirmed that colloidal lipid particles with the average size of 150 nm stabilized Pickering emulsion had a similar oxidation rate as conventional sodium caseinate–stabilized emulsions [17]. Thus, the effects of the particles size or thickness of the interfacial layer in Pickering emulsion on lipid oxidation are largely unexplored. Moreover, the effects may be system-dependent.

Adding an antioxidant to the emulsion is certainly an effective method to limit the oxidation. Free radical scavenging antioxidants are ordinarily utilized species for the control of lipid oxidation in oil-in-water emulsions. Free radical scavengers inhibit lipid oxidation by rapidly reacting with lipid radicals and forming low-energy antioxidant radicals that are ineffective in promoting fatty acid oxidation [18]. As the oil–water interface is where the lipid oxidation initiates and propagates, the efficiency of an antioxidant in surfactant-based emulsions depends on its concentration in the interfacial region [19,20,21]. Applying the pseudophase kinetic model, Losada-Barreiro et al. have found that, when increasing the surfactant volume fraction, there is much more antioxidant distributed in the interfacial region, but at the same time, the interfacial volume (Φ_I_, defined hereafter as Φ_I_ = V_surfactant_/V_emulsion_) is increased, leading to the dilution of antioxidants in that region and thus the decrease in their efficiency. Thus, modulating the interfacial concentration of antioxidant in emulsions is also a worth-trying strategy in improving the antioxidant behavior and retarding the oxidation in emulsions. Although the effects of various fabrication parameters, such as pH, temperature, emulsifier concentration, etc., on the distribution behaviors of various antioxidants in surfactant based emulsions have been reported [22,23], the antioxidant distribution in particles involving emulsions have rarely been studied.

As an amphiphilic prolamin, zein has distinct hydrophobic and hydrophilic domains and can self-assemble into nanoparticles. So far, the most straightforward and reproducible method for the preparation of zein nanoparticles is via the anti-solvent precipitation. With fine-tuning of on experiment parameters, different sizes of zein nanoparticles could be fabricated [24]. Theoretically, the thickness of interfacial layer of zein stabilized Pickering emulsion could then be modulated. However, the interfacial loading efficiency of zein nanoparticles is low. Pickering emulsions stabilized solely by zein nanoparticles are easily subjected to creaming and oiling, and instead of forming fluid-like emulsions, gelled-like emulsions are usually obtained [25]. The development of particles and surfactant co-stabilized emulsion systems has been emerging as a research spot recently. Tween 20, a nonionic water-soluble surfactant, is widely used in the food industry, and it has been reported that Tween 20 may bind with zein molecules to form zein/Tween 20 complexes driven by hydrogen bonding and hydrophobic interactions, which improved the surface activity of zein nanoparticles [26,27]. Besides, the addition of different amounts of Tween 20 provided a variable interfacial volume fraction (Φ_I_ = V_surfactant_/V_emulsion_, varied from 0.005 up to 0.04), which enabled the application of the pseudophase kinetic model in determining the interfacial concentration of antioxidant in emulsion system.

In our previous study, a Pickering emulsion co-stabilized by zein nanoparticles and Tween 20 (ZPE) was fabricated, and it performed better chemical stability than Tween 20 stabilized emulsions [28]. We also found that the addition of zein nanoparticles affected the distribution of GA and enhanced its antioxidant efficiency. Considering the fact that the particle size of zein nanoparticles can be finely tuned, this system would thus serve as an ideal system for exploring the effects of particle size on the interfacial concentration of antioxidants and the oxidation stability of emulsions.

In this paper, different sizes of zein nanoparticles were fabricated to engineer the interfacial layer of emulsions. The antioxidant activity of GA in zein nanoparticle solutions (70, 130, and 220 nm) was carried out by determining the 2,2-Diphenyl-1-picrylhydrazyl (DPPH) scavenging activity. The interfacial concentration of GA in emulsions was determined by employing a pseudo-phase kinetic model, and the oxidative stability of the Pickering emulsion, with or without gallic acid, was assessed by using the accelerated oxidation test. How the particle size modulated the interfacial concentration of GA in ZPEs and their oxidative stability were investigated. This research extends our current knowledge on how interfacial engineering manipulates the interfacial concentration of antioxidant and then affect the oxidative stability of emulsions.

## 2. Materials and Methods

### 2.1. Materials

Zein (98%) was purchased from Wako Pure Chemical Industries Co., Ltd. (Wako Inc., Osaka, Honshu, Japan). Stripped corn oil and N-(1-naphthyl) ethylenediamine (98%) were obtained from Shanghai Aladdin Bio-Chem Technology Co., Ltd. (Aladdin Inc., Shanghai, China). Gallic acid (Da Mao Inc., Tianjin, China) and Tween 20 (Kermel Inc., Tianjin, China) were of the highest purity available and used as received. The 4-Hexadecylaniline (97%) was bought from Shanghai Aladdin Bio-Chem Technology Co., Ltd. (Aladdin Inc., Shanghai, China), for the synthesis of 4-Hexadecylbenzenediazonium tetrafluoroborate (16-ArN_2_BF_4_) by diazotization, on the basis of a published method [29]. Double-distilled water was used throughout the experiments, and other reagents used were of analytical grade.

### 2.2. Fabrication of Zein Colloidal Particles with Different Sizes

Different sizes of zein nanoparticles were fabricated, using a typical anti-solvent precipitation, method with some modifications [24]. Size-controlled zein nanoparticles was achieved by modulating experiment parameters such as a mixing-sequence, the ratio between ethanol and water, zein concentration, etc. For instance, when preparing zein nanoparticles with the (number) averaged particle size of 70 nm with a narrow size distribution, zein powder (1.0 g) was dissolved in 40 mL of 70 v/v% aqueous alcohol solution, to yield a zein stock solution. The stock solution was then syringed into a beaker filled with 120 mL double-distilled water, under continuous shearing (10,000 rpm) (IKA T18 digital ULTRA-TURRAX homogenizer, IKA Inc., Staufen Im Breisgau, Baden-Württemberg, Germany). The duration of the mixing stage was controlled strictly at 5 min. Ethanol was removed under reduced pressure by rotary evaporation, until zein dispersion was concentrated to 50 mL. Finally, the dispersion was centrifuged for 10 min at 880 g, to remove large aggregates. In a similar way, 4.0 g of zein powder was dissolved in 40 mL of 90 v/v% aqueous alcohol solution, which was then syringed into a beaker filled with 120 mL double-distilled water, under continuous shearing (10,000 rpm), to fabricate zein nanoparticles with an average particle size of 130 nm. As for the fabrication of zein nanoparticles of 220 and 300 nm, 120 and 65 mL of double-distilled water was syringed into the zein stock solution (2.0 g zein powder dissolving in 40 mL of 90 v/v% aqueous alcohol solution), under continuous shearing (10,000 rpm), respectively. Ethanol was removed under reduced pressure by rotary evaporation, until zein dispersion was concentrated to 50 mL. Finally, the dispersion was centrifuged for 10 min at 880 g, to remove large aggregates. The concentration of the as-prepared zein nanoparticles with different sizes was determined by the Lowry’s method and then diluted to 1 wt.%, using double-distilled water for further experiments. The as-prepared zein nanoparticles dispersions with the zeta-potential above +35 mV could be stored in the refrigerator at 4 °C, over one month, without sedimentation.

### 2.3. Particle Size, Polydispersity Index (PDI), and Ζ-Potential Measurement

Particle size, PDI, and ζ-potential were measured, using a Nanosizer ZS 90 instrument (Malvern Inc., Worcestershire, UK). All samples were diluted 20 times, using 0.04 M acetic acid solution to pH = 3.0, before measuring. All measurements were conducted in duplicate, at 25 °C.

### 2.4. Preparation of Pickering Emulsion Co-Stabilized by Zein Nanoparticles and Tween 20 (Zpes)

The water phase of the prepared ZPEs included zein nanoparticles, Tween 20 with or without GA, while the oil phase was stripped corn oil. Specifically, during the preparation process, zein nanoparticles with different average particle size were set at the concentration of 1 wt.%, in the continuous phase. The volume ratio of the water phase to oil phase was set at 5:5; the total emulsion was 10 mL. Gallic acid and Tween 20 were added into the aqueous phase, prior to the preparation of emulsions. Stripped corn oil was slowly added to the zein nanoparticles solution in a glass vial, under 10,000 rpm shearing (IKA T18 digital ULTRA-TURRAX homogenizer, IKA Inc., Staufen Im Breisgau, Baden-Württemberg, Germany), for 2 min, at room temperature, to prepare the Pickering emulsions. The concentration of GA in the final emulsion was adjusted to 0.5 mM. When determining the k_obs_ values and (GA_I_) in ZPE by pseudophase kinetic method, the concentration of Tween 20 in the emulsion ranged from 0.5% to 4% (v/v). Herein, k_obs_ is the observed first order rate constant of the reaction between 16-ArN_2_^+^ and the GA in emulsions. (GA_I_) is the interfacial concentration of GA and the percentage of GA in the interfacial region is %GA_I_. Moreover, the concentration of Tween 20 was set at 0.5% (v/v) when studying the oxidative stability, the microstructure, and the surface loading content of zein nanoparticles in ZPEs. Photographs of emulsion were taken with a Canon digital camera (Guangzhou, China).

### 2.5. Determination of K_obs_ Values and (GA_I_) in ZPE

To determine the distribution and interfacial concentration of GA, without causing destruction to the intact emulsion, a chemical kinetic method was used [21]. ZPEs stabilized by different sizes of zein nanoparticles (70, 130, and 220 nm) with the concentration of 1 wt.% and Tween 20 (0.5%, 1%, 2%, and 4%) respectively, were used for the determination of k_obs_ values and GA_I_%. Tween 20 (0.5%, 1%, 2%, and 4%) stabilized emulsions were set as the control. The concentration of GA added in the final emulsions was 0.5 mM. The system of ZPEs and the surfactant-based emulsions were divided into three regions (aqueous, interface, and oil), where gallic acid distributed. This kinetic method associated the partition constants of antioxidants (P_W_^I^: partition constant between the water and interfacial regions, P_O_^I^: partition constant between the oil and interfacial regions) with the observed rate constant between GA and 16-ArN_2_^+^, k_obs_. The chemical probe 4-hexadecylbenzenedizonium ions, 16-ArN_2_^+^, which is oil-insoluble and water-insoluble, only reacts in the interfacial region of the emulsion with the antioxidants. Thus, the k_obs_ values of the reaction between GA and the 16-ArN_2_^+^ could be applied to obtain the P_W_^I^ of GA in ZPEs by the azo dye derivatization method. The reaction was initiated by adding an aliquot (40 μL) of a 0.17 M 16-ArN_2_^+^ stock solution into emulsions. At a specific time, 200 μL of emulsion mixture that contained unreacted 16-ArN_2_^+^ was added to a tube with 2 mL NED (0.02 M). NED referred to as the solution of N-(1-naphthyl) ethylenediamine (Aladdin Inc., Shanghai, China) dissolved in a 50:50 (v/v) BuOH/EtOH mixture. After 20 min of stirring, the tube was centrifuged for 2 min, at 5500 g, and the optical density (OD) values of supernatant at 572 nm were collected by using a Multimode Plate Reader (PerkinElmer Inc., Waltham, MA, USA). As 16-ArN_2_^+^ reacts with NED much more rapidly than with GA; the concentration of unreacted 16-ArN_2_^+^ is proportional to the OD value of the azo dye at λ = 572 nm. Then, values of k_obs_ were obtained by fitting the OD value vs. time data to the integrated first-order rate equation via a non-linear least squares method. As extremely hydrophilic the GA is, only the partition constant between the water interfacial, P_W_^I^, which is defined by Equation (1), is needed to describe its distribution. The addition of different concentrations of Tween 20 provided a variable k_obs_ values versus interfacial volume fraction (Φ_I_) for the application of the pseudophase model, to determine the P_W_^I^ values:

(1) $$P_{W}^{I}=\frac{{\mathrm{GA}}_{I}}{\left({\mathrm{GA}}_{W}\right)}$$

(2) $$k_{\mathrm{obs}}=\frac{[{\mathrm{GA}}_{T}]k_{I}P_{W}^{I}}{\Phi_{I}P_{W}^{I}+\Phi_{W}}$$

(3) $$\frac{1}{k_{\mathrm{obs}}}=\frac{\Phi_{W}}{k_{I}[{\mathrm{GA}}_{T}]P_{W}^{I}}+\frac{\Phi_{I}}{k_{I}[{\mathrm{GA}}_{T}]}$$

Equations (2) and (3) describe the relationship between P_W_^I^ and k_obs_, where [GA_T_] is the GA concentration in total emulsion volume; k_I_ indicates a second-order rate constant in interfacial region; and Φ_w_ is the volume fraction of the aqueous phase. Readers may refer to Reference [30] for detailed calculations. Moreover, the partition constants P_W_^I^ could be assessed by fitting the experimental data to Equation (3) (1/k_obs_ vs. Φ_I_).

Then, determining the percentage of GA in the interfacial region (GA_I_%) could be obtained straightforward, by Equation (4), and the interfacial concentration of GA, (GA_I_), was calculated by employing Equation (5):

(4) $${\mathrm{GA}}_{I}\%=\frac{100\Phi_{I}P_{W}^{I}}{\Phi_{I}P_{W}^{I}+\Phi_{W}}$$

(5) $$\left({\mathrm{GA}}_{I}\right)=\frac{\left[{\mathrm{GA}}_{T}]({\mathrm{GA}}_{I}\%\right)}{\Phi_{I}}$$

### 2.6. Oxidative Stability and Physical Stability of Emulsions

To study the physical stability of ZPEs, an accelerated oxidation test was carried out. ZPEs with GA (0.5 mM) added, stabilized by zein nanoparticles (70, 130, 220 nm), were prepared and allowed to spontaneously oxidize at 55 °C in the dark. The creaming index and oiling phenomenon were then recorded after two weeks’ accelerated oxidation test. To study the effect of (GA_I_) on the ZPE oxidative stability, ZPEs with GA (0.5 mM) added, stabilized by zein nanoparticles (70, 130, and 220 nm), were prepared and allowed to spontaneously oxidize at 55 °C, in the dark, for further measurement. Then, the primary oxidation products of ZPEs were determined by the ferric thiocyanate method every other day, to evaluate their oxidative stability. Moreover, ZPEs without GA addition, stabilized by zein nanoparticles (70, 130, and 220 nm), were prepared to study the effect of particle size on the ZPE oxidative stability. Emulsion with or without GA addition, stabilized only by 0.5% Tween 20 (T20), was selected as a control. Their hydroperoxide was measured every other day, by adding 0.3 mL of emulsions to 1.5 mL isooctane/2-propanol (3:1, v/v) under vortex for 10 s (3 times). The mixture was then isolated by centrifugation at 5500 g for 5 min, and the organic supernatant (200 µL), 50 µL of 3.97 M ammonium thiocyanate, and 50 µL of ferrous iron solution (prepared by mixing 0.132 M BaCl_2_ and 0.144 M FeSO_4_) were added, in turn, to the 2.8 mL of methanol/1-butanol (2:1, v/v) solution. After mixing and stored for 20 min, the absorbance of the solution was measured at 510 nm. Hydroperoxide concentrations were determined by using a standard curve made from cumene hydroperoxide. All measurements were taken three times.

### 2.7. Evaluation of Antioxidant Capacity

The evaluation of antioxidant capacity was carried out by determining the 2,2-Diphenyl-1-picrylhydrazyl (DPPH) scavenging activity. A fixed concentration at 1 wt.% of zein nanoparticle solutions (70, 130, and 220 nm), with or without GA (0.5 mM), were prepared, in order to react with 1.0 mL DPPH methanol solution (0.33 mM), and they were allowed to react for 30 min at ambient temperature. An aliquot of 200 µL of the resultant mixture was then transferred to a 96-well microplate, and the absorbance was measured at 517 nm. The DPPH scavenging activity was calculated as follows:

DPPH radical scavenging activity (%) = (A_blank_ − A_sample_)/A_blank_ × 100 (6)

where A_blank_ is the control reaction without GA and zein particles, and A_sample_ is the absorbance of the test sample.

### 2.8. Microstructure of Zpes Revealed by Confocal Laser Scanning Microscope (CLSM)

The micro-structure of ZPEs was observed with a Leica TCS SP5 confocal laser scanning microscope (Leica Microsystems Inc., Heidelberg, Baden-Württemberg, Germany). ZPEs were dyed with a mixed fluorescent dye solution, consisting of 0.1 mg/mL Nile red to stain oil and 0.1 mg/mL Nile blue A to stain zein nanoparticles. The stained emulsions were placed on concave slides and covered with coverslips and observed with a 10× lens and an argon krypton laser, with an excitation line of 488 nm and a helium neon laser (He/Ne) with excitation at 568 nm.

### 2.9. Determination of Surface-Loading Content of Zein Nanoparticles in ZPE (Γ)

The loading content of zein nanoparticles at the surface of droplets in ZPEs stabilized by different sizes of zein nanoparticles was quantified according to a previous study, with modification [31]. The freshly prepared ZPEs were then centrifuged at 100 g for 20 min. Then the serum phase was filtered with a 0.45 μm membrane filter. The protein concentration in the serum phase was determined by the Lowry method, and the surface protein loading (Γ) (mg/m^2^) was calculated by the following equation [31]:

Γ(mg/m^2^) = (C_t_ − C_s_) × D_3,2_/6Φ (7)

where C_t_ (mg/mL) is the initial protein concentration in the aqueous phase, and C_s_ (mg/mL) is the concentration of protein in the serum phase. D_3,2_ (μm) is the surface average diameter of emulsion droplets determined by using a Malvern MasterSizer 3000 (Malvern Inc., Worcestershire, UK), and Φ represents the volume fraction of oil.

### 2.10. Thermodynamic Parameters of the Binding Process Quantified by the Isothermal Titration Calorimetry (ITC)

The thermodynamic parameters of the binding process between zein nanoparticles and GA at pH 3.0 were measured following the previous publication by ITC 200 microcalorimeter (MicroCal Inc., Northampton, UK) at 25 °C. Zein nanoparticles solution (10 mg/mL) and GA solution (50 mM) were prepared according to the method described previously [28,32]. Zein and GA solutions were placed in 200 μL of reaction cell and 39.4 μL syringe, respectively. The acetic acid solution (0.04 M) was used as a blank. The titration was performed with 19 successive 2 μL injections of GA solution, while the first injection was 0.4 μL and not collected into the final result. Each addition lasted 2 s, with an interval of 180 s between consecutive injections. The stirring speed was set at 800 rpm. The number of binding sites (N), affinity constant (K), enthalpy change (ΔH), and entropy change (ΔS) were calculated.

### 2.11. Statistical Analysis

The reaction between 16-ArN_2_^+^ and NED was monitored for 2–3 t_1/2_, with typical correlation coefficients >0.995 in triplicate experiments, given k_obs_ values within 7–9%. SPSS 21.0 software was applied for statistical analysis by one-way analysis of variance (ANOVA, with Tukey’s HSD multiple comparison), with the level of significance set at *P* < 0.05. Data are presented as mean values ± standard deviation.

## 3. Results and Discussions

### 3.1. Characterization of ZPEs

**Figure 1:**
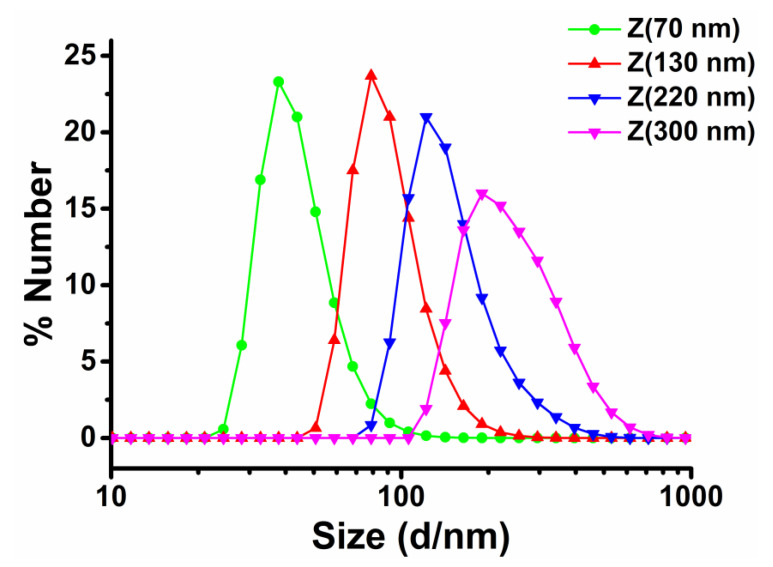
Distribution of zein nanoparticles at different sizes: 73.52 ± 0.02 nm, 131.30 ± 0.70 nm, 218.2 ± 1.44 nm, 300.4 ± 2.58 nm, *P* < (0.05). PDI = 0.167, 0.111, 0.108, 0.125, respectively.

**Figure 2:**
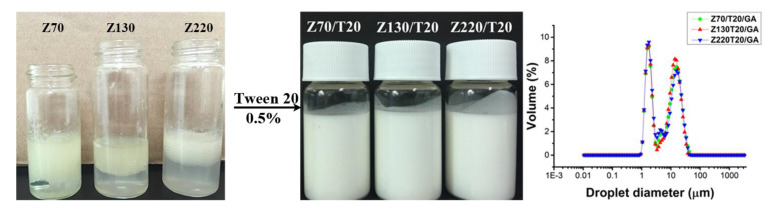
Photograph of emulsions stabilized by different sizes of zein nanoparticles (1 wt.%), with or without the addition of Tween 20 (0.5%), at pH 3.0; and droplet size distribution of ZPEs.

Particle size of zein nanoparticles could be finely tuned by adjusting the fabrication parameters, such as the concentration of zein in ethanol solution, the volume ratio of solvent/anti-solvent, the stirring rate, and during the anti-solvent precipitation process [24]. Thus, by manipulating the fabricating parameters, size-controlled zein nanoparticles were achieved. The number mean diameter of zein nanoparticles was tested in this study, and the polydispersity index was used to describe the degree of non-uniformity of the size distribution of zein nanoparticles. As shown in Table 1, the mono-dispersed zein nanoparticles with the size distribution of 73.52 ± 0.02 nm, 131.30 ± 0.70 nm, 218.2 ± 1.44 nm, and 300.4 ± 2.58 nm were named Z (70 nm), Z (130 nm), Z (220 nm), and Z (300 nm), respectively (Figure 1). The polydispersity index (PDI) of all zein nanoparticles within 0.2 was considered to be a homogenous monodisperse dispersion, without significant aggregation. All zein nanoparticles showed ζ-potential values over +30 mV, indicating a strong charge repulsion and thus great colloidal stability. As shown in Figure 2, zein nanoparticles could not form stable oil in water Pickering emulsion solely, but with the addition of Tween 20 (0.5% v/v), the physical stability of emulsions improved and fully emulsified emulsion systems were obtained. The emulsion droplet size distributions of ZPEs were presented in Figure 2. The small emulsion droplets with the averaged droplet size of 1 μm were more likely to be stabilized only by Tween 20, and larger emulsion droplets with the averaged droplet size of 20 μm should be co-stabilized by zein nanoparticles and Tween 20. The fact that the determined values of D_3,2_ (Table 1) were closer to the second peak suggested that the emulsion systems were dominated by the Pickering-type stabilization. We also noticed that larger zein nanoparticles led to smaller D_3,2_ of ZPE (Table 1), and the increase in the efficiency of the surfactant to stabilize the interface as more space was available between the largest nanoparticles might serve as a possible explanation. Moreover, during the two weeks’ accelerated oxidation test under 55 °C, the creaming index of ZPEs stabilized by different sizes of zein nanoparticles were all within 30%, with no oil leakage. Compared to surfactant-based emulsions, most Pickering emulsions are easily subjected to creaming because of their large droplet size [10,13], but they performed better physical stability against coalescence and oiling than the surfactant-based emulsion. Thus, the oxidative stability of ZPEs still could be studied in such systems, though creaming occurred. In our previous study [28], we found that excessive Tween 20 (4% v/v) would replace zein nanoparticles and dominate the interface. However, at the volume fraction of 0.5%, Tween 20 facilitated the zein nanoparticles to stabilize Pickering emulsion. However, when the mean particle size of zein reached 300 nm, severe sedimentation occurred after centrifugation, and the sedimentation also appeared when preparing the emulsion. Thus, only Z (70 nm), Z (130 nm), and Z (220 nm) were utilized for the following experiments.

### 3.2. Effect of Particle Size on Oxidative Stability of ZPE

**Figure 3:**
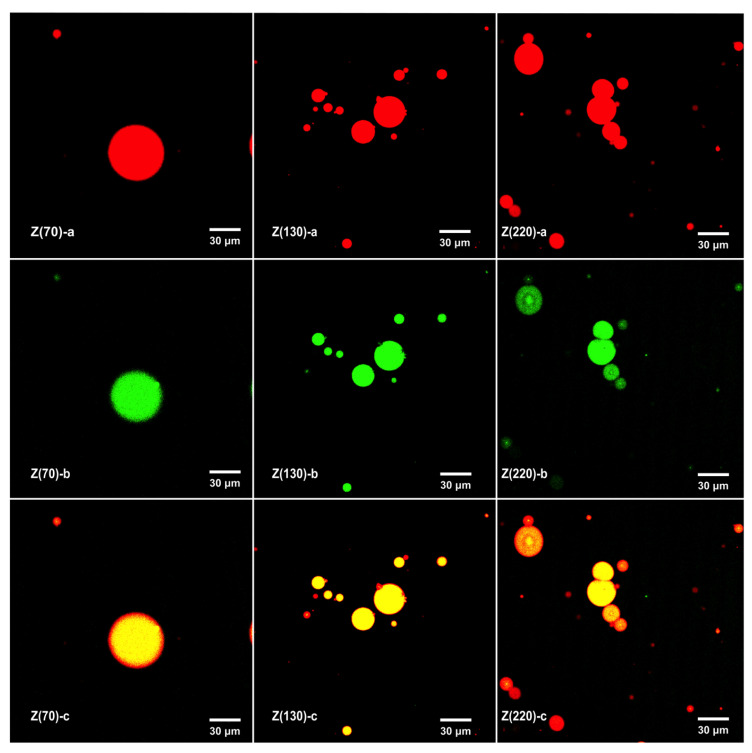
CLSM images of the ZPEs stabilized by different size of zein nanoparticles (70, 130, and 220 nm) with 0.5% Tween 20. Protein was stained red by Nile blue A (**a**), and the oil phase was stained green by Nile red (**b**), (**c**) was combined image of a and b.

**Figure 4:**
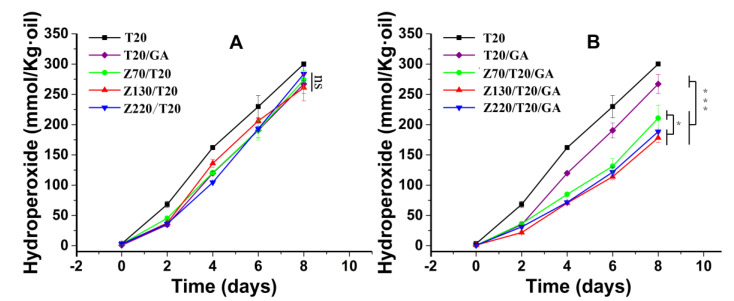
Formation of lipid hydroperoxides in ZPEs with (**A**) or without GA (0.5 mM) (**B**) in eight days, at a 0.5% volume fraction of Tween 20 (T20) and zein (1 wt.%) with different particle sizes (70, 130, 220 nm). Z(70)/T20/GA, Z(130)/T20/GA, Z(220)/T20/GA represented the presence of GA (0.5 mM) in these ZPEs, and the average particle size of zein nanoparticles was 70, 130, 220 nm, respectively. Z(70)/T20, Z(130)/T20, Z(220)/T20 represented the absence of GA, and the average particle size of zein nanoparticles was 70, 130, 220 nm, respectively. The emulsion stabilized only by Tween 20 was set as the control. Data were presented as the mean value ± SD (n ≥ 3): (ns) *P* > 0.05, (*) *P* < 0.05, (**) *P* < 0.01, and (***) *P* < 0.001.

As shown in Figure 3, zein nanoparticles were stained by Nile blue A, and the oil phase was stained by Nile red. The oil droplets were surrounded by zein nanoparticles, confirming the formation of a nanoparticle interfacial layer in ZPEs. The D_3,2_ (Table 1) and polydispersity (Figure 2) of emulsions stabilized with different nanoparticle sizes were similar, indicating the influence from emulsion droplet size could be excluded when studying the effects of the particle size on the oxidative stability of emulsions. To study the effect of particle size on oxidative stability of ZPEs, the generation of hydroperoxide was recorded in Figure 4A. The formation of hydroperoxide among ZPEs stabilized by Z (70 nm) + 0.5% Tween 20 (Z70/T20), Z (130 nm) + 0.5% Tween 20 (Z130/T20), and Z (220 nm) + 0.5% Tween 20 (Z220/T20), without the addition of GA, showed insignificant different (*P* > 0.05). ZPEs stabilized by different sizes of zein nanoparticles performed unequal interfacial thickness; however, unlike some studies reporting that thicker interface layer retarded lipid oxidation more effectively [1,33], at a fixed concentration, different sizes of zein nanoparticles had a similar effect on the oxidative stability. This could be explained by the fact that zein nanoparticles were not homogeneously, but porously, absorbed at the droplet surface, which allowed the diffusion of small molecular pro-oxidant and peroxyl radical in the interfacial region. This suggested that thicker interface layers did not guarantee better oxidation stability, which was consistent with the report by Schröder et al. [17]. Schröder et al. found that colloidal lipid particles stabilized Pickering emulsion exhibited a similar oxidation rate as conventional sodium caseinate-stabilized emulsion. It was worth noting that the physical barrier effect of zein nanoparticles did play a part in delaying the lipid oxidation. Though the DPPH radical scavenging capacity of GA was much higher than that of the zein nanoparticles (Table 2), emulsions co-stabilized by zein nanoparticles and 0.5% Tween 20 showed similar oxidation stability as the emulsion stabilized by 0.5% Tween 20 with the addition of 0.5 mM GA.

In the presence of GA (Figure 4B), ZPEs with different sizes of zein nanoparticles showed different oxidative stabilities: Z130/T20/GA > Z220/T20/GA > Z70/T20/GA. Z130/T20/GA produced lower concentration of hydroperoxide than that of Z220/T20/GA, but did not reach a significant level at *P* < 0.05. However, both Z130/T20/GA and Z220/T20/GA showed stronger oxidative stability than Z70/T20/GA, *P* < 0.05. All ZPEs showed stronger oxidative stability than emulsions stabilized only by Tween 20 with GA (T20/GA), *P* < 0.001. In this case, both the physicochemical property of zein nanoparticles at the interface and the interfacial concentration of GA, (GA_I_), affected the emulsion oxidation. We found that zein nanoparticles showed a weak radical scavenging capacity because of the exposure of the originally buried antioxidant amino acid residues under the measurement conditions. Nevertheless, though zein exhibited antioxidant capacity to some extent, the antioxidant capacity of zein nanoparticles (1 wt.%) was negligible as compared to that of the GA at 0.5 mM. As shown in Table 2, the radical scavenging capacity of blank zein nanoparticle was around 20%, but GA with or without zein nanoparticle were both around 95%. Thus, particle size of zein nanoparticles had negligible effect on their antioxidant capacity, and the antioxidant effect rooting from zein nanoparticles could be excluded. The thickness of interface layer was also not taken into account, because we have proven that thicker zein nanoparticle layer did not serve as a better protection layer. Consequently, the (GA_I_), which could be tuned by the size of zein nanoparticles, might be the dominant factor that determines the oxidative stability.

### 3.3. Effect of Particle Size on (GA_I_) of ZPE

**Figure 5:**
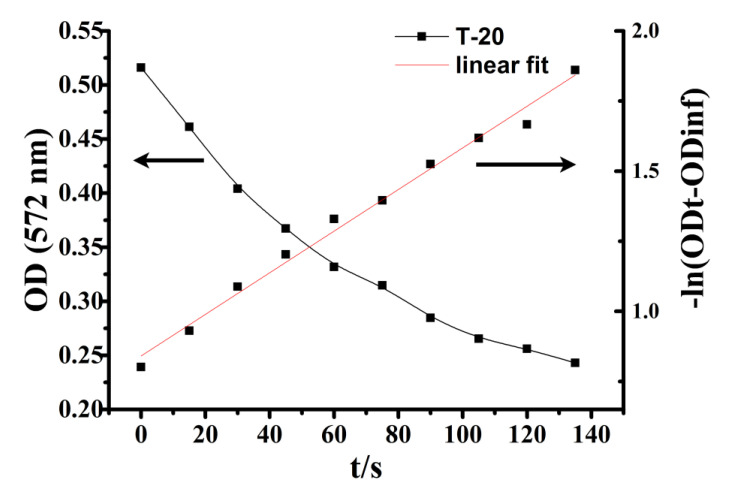
Examples of the determination of k_obs_ for the reaction between 16-ArN_2_^+^ and GA in emulsion stabilized by 0.5% Tween 20 (T20) without zein nanoparticles added, 1:1 oil to water ratio composed of stripped corn oil, showing the variation of the absorbance of the azo dye with time and the corresponding ln plot; [16-ArN_2_^+^] ≈ 3.3 × 10 ^−4^ M, [GA] = 5 mM, at room temperature.

**Figure 6:**
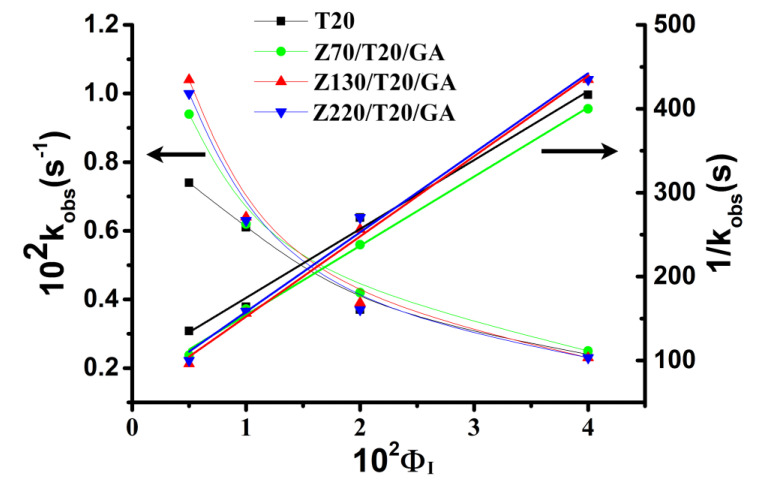
Variation of k_obs_ values and 1/k_obs_ for the reaction between 16-ArN_2_^+^and GA in emulsions. The solid lines are the theoretical curves achieved by fitting the experimental data to Equations (6)–(8) and their reciprocals.

**Figure 7:**
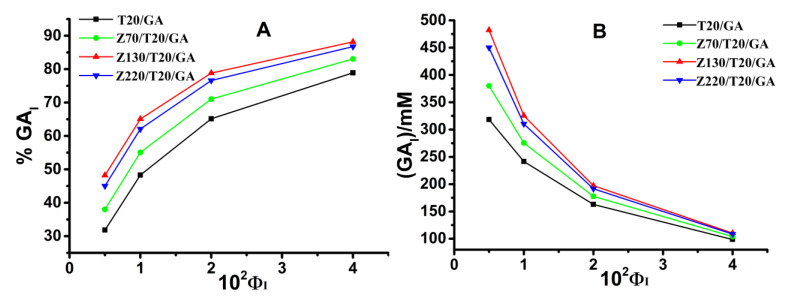
GA_I_% (**A**) and (GA_I_) (**B**) of ZPEs with different sizes of zein nanoparticle (1 wt.%). The fractions of Tween 20 varied from 0.005 to 0.04.

Gallic acid is a water-soluble antioxidant and almost insoluble in oil [34]. At a fixed volume of emulsifier in emulsion, the higher the percentage of GA at the interface, the more efficient the antioxidant capacity is, since antioxidant efficiency is positively correlated with their fractions in the interfacial region. Thus, the mechanism of how antioxidant distributes in the Pickering emulsion is vital for improving the antioxidant efficiency. We hypothesized that the variation in the size of zein nanoparticles would affect the oxidative stability via modulating the interfacial concentration of GA. Therefore, we determined the distribution of GA in the intact emulsions and analyzed how the size of zein nanoparticles affected the concentration of GA in the interfacial region. The distribution of GA in ZPEs was determined by the pseudophase model. In a typical experiment, the variation of the absorbance of the azo dye with time and the corresponding ln (OD _t_ – OD _inf_) plot versus time were presented in Figure 5. The value of k_obs_ (k_obs_ is the observed rate constant for the reaction of the chemical probe 16ArN_2_^+^ with GA) was then obtained by fitting the OD value vs. time data to the integrated first-order rate equation via a non-linear least squares method. In this way, k_obs_ values of ZPEs with interfacial volume fraction (Φ_I_) varying from 0.005 to 0.04 were also obtained. The linear fitting of 1/k_obs_ versus Φ_I_ in Figure 6 was then used to calculate the values of P_W_^I^ (the partition constant between the water and interface region) by Equations (2) and (3), which were summarized in Table 1. The increase in P_W_^I^ was a strong indicator of elevated interfacial concentration of GA. The interfacial percentage and the interfacial concentration of GA (GA_I_% and (GA_I_)) were calculated by employing the Equations (4) and (5), respectively, and were presented in Figure 7. Though the GA_I_% was increased when increasing the Φ_I_ (Figure 7A), (GA_I_) decreased as Φ_I_ increased from 0.005 to 0.04 (Figure 7B) due to the dilution effect [21]. As shown in Figure 7B, at a fixed Φ_I_ = 0.005, different sizes of zein nanoparticle led to different (GA_I_), suggesting that the size of nanoparticles did affect the interfacial concentration of GA, though the effect of nanoparticle size from 130 to 220 nm is not significant. Interestingly, the (GA_I_) showed neither positive nor negative correlation with the increasing particle size, suggesting particle size was not the direct and dominant factor regulating the (GA_I_).

**Figure 8:**
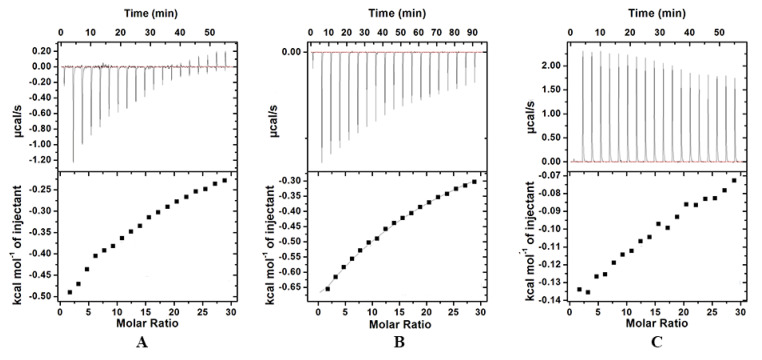
Thermograms (top panels) and binding isotherms (bottom panels) corresponding to the titration of the zein nanoparticles (**A**): Z(70 nm), (**B**): Z(130 nm), and (**C**): Z(220 nm) with GA solutions at pH 3.0.

In our previous study, zein nanoparticles could bind with GA and increase the (GA_I_). Thus, different sizes of zein nanoparticles might exhibit different affinities to GA, which may then affect (GA_I_). To test and verify the hypothesis, the affinities between GA and different size of zein nanoparticles were determined by using isothermal titration calorimetry (ITC). The heat flow versus time profiles from the titration of GA with different sizes of zein nanoparticles (1 wt.%) at pH 3.0 are shown in Figure 8. At each GA injection, the heat exchange of each peak in the calorimeter cell containing zein nanoparticles was recorded. As ΔH < 0 and ΔS < 0, the interaction between zein nanoparticles with GA was mainly attributed to the hydrogen bonding and van der Waals forces [32,35,36]. In Table 3, the values of Ka (the association equilibrium constant) <33 M^−1^ and N (the binding-site number) < 0.2 indicated that the interaction between zein nanoparticles and GA was weak, consisting with our previous report [28]. Though the Ka value of Z(220 nm) (18 M^−1^) was smaller than that of Z(70 nm) (32.0 M^−1^) and Z(130 nm) (32.2 M^−1^), the (GA_I_) among ZPEs actually followed the sequence of ZPEs stabilized by Z130/T20/GA > Z220/T20/GA > Z70/T20/GA (Figure 7B). Consequently, the difference in binding affinities between GA and different sizes of zein nanoparticles actually had limited influence on the regulation of (GA_I_).

In our previous work, we found that, upon increasing the concentration of zein nanoparticles, the loading of particles at the interface was increased and further lifted the (GA_I_). In this paper, the interfacial loading content of zein nanoparticles of ZPEs was collected by the Lowry method and presented in Table 1. Our result showed that Γ in these ZPEs followed the sequence of Z130/T20/GA > Z220/T20/GA > Z70/T20/GA. The result suggested that the loading content of zein nanoparticles at the interface (Γ) was affected by the nanoparticles’ size. A possible reason might be the variation of wettability among zein nanoparticles with different sizes. As it was reported in Pickering emulsions stabilized by sweet potato and corn starch nanoparticles, the particle size ranging from 100 to 220 nm exhibited higher interfacial loading efficiency than those with a particle size less than 100 nm or more than 220 nm, due to the difference in particle wettability [37]. Moreover, the sequence of (GA_I_) (Figure 7B) was consistent with the sequence of Γ (Table 1), which again confirmed that the (GA_I_) had a positive correlation with the interfacial loading content of zein nanoparticles. This could be due to the fact that GA bonds with zein nanoparticles not only in the interfacial region but also in the bulk water phase, and the increase in the ratio of interfacial loaded zein nanoparticles to the bulk dispersed zein nanoparticles lifted the interfacial concentration of GA. That was why the difference in affinities was not the direct regulation factor on the (GA_I_), as compared to the interfacial content of zein nanoparticles. Furthermore, (GA_I_) in ZPEs followed the sequence of ZPEs stabilized by Z130/T20/GA > Z220/T20/GA > Z70/T20/GA > T20/GA, which was the same as the sequence of the oxidative stability (Figure 4). Thus, it could be concluded that a higher Γ, which could be achieved by altering the nanoparticle size, increased the (GA_I_) and further improved the oxidative stability of ZPEs. Consequently, rather than modulating the thickness of interfacial layer, increasing the concentration of antioxidant at the interface by maximizing the interfacial loading of zein nanoparticles should be a better solution to retard the oxidation rate in the Pickering emulsion.

## 4. Conclusions

In this paper, a novel Pickering emulsion was prepared, using zein nanoparticles and Tween 20 as stabilizers. Tween 20 (0.5% v/v) facilitated the interfacial absorption of zein nanoparticles, which enhanced the physical stability of ZPEs. Our study revealed that the thickness of the interfacial nanoparticle layer exerted non-direct influence on the oxidative stability. In the absence of GA, there was no significant difference in the production of hydroperoxide among emulsions stabilized with zein nanoparticles of different particle size distributions. However, the physical barrier effect of zein nanoparticles did play a part in delaying the lipid oxidation. When adding GA (0.5 mM) in ZPEs, higher interfacial concentration of GA, (GA_I_) and stronger oxidative stability were observed in ZPE stabilized by zein nanoparticles, with the average particle size of 130 nm, than those stabilized by zein nanoparticles of 70 and 220 nm. It was because GA could bind with zein nanoparticles via hydrogen bonding, and the (GA_I_) had a positive correlation with the interfacial loading content of zein nanoparticles, which was affected by the particle size. In this case, the (GA_I_) governed the oxidative stability of ZPEs. Compared to thickening the interfacial layer, manipulating the interfacial distribution of antioxidant by interfacial engineering is a more efficient alternative way to prevent lipid oxidation. It is worth putting more efforts into the design of the Pickering emulsion interface, for better oxidative stability.

## Figures and Tables

**Table 1:** Particle size of zein nanoparticles, the surface-averaged mean diameter (D _3,2_), the interfacial loading content of zein nanoparticle (Γ), and P_W_^I^ determined in intact ZPEs (Φ_I_ = 0.005).

	Size (d·nm)	D _3,2_ (μm)	Γ (mg/m ^2^)	P_W_^I^
Z (70 nm)	73.52 ± 0.02	9.99 ± 0.03	0.58 ± 0.03	61.31
Z (130 nm)	131.30 ± 0.70	9.77 ± 0.22	0.77 ± 0.02	93.12
Z (220 nm)	218.2 ± 1.44	9.62 ± 0.18	0.71 ± 0.03	81.85

**Table 2:** DPPH radical scavenging capacities of zein nanoparticles with or without GA addition.

Zein Nanoparticles	DPPH Radical Scavenging Activity% (30 min)	0.5 mm GA + Zein Nanoparticles	DPPH Radical Scavenging Activity% (30 min)
Tween 20	—	Tween 20	95.00 ± 0.47
Z (70 nm)	20.94 ± 1.37	Z (70 nm)	94.25 ± 0.26
Z (130 nm)	22.64 ± 0.76	Z (130 nm)	95.87 ± 0.36
Z (220 nm)	21.82 ± 1.37	Z (220 nm)	94.74 ± 0.57

**Table 3:** The number of binding sites (N), affinity constant (K), enthalpy change (ΔH), and entropy change (ΔS) of the binding between zein nanoparticles and GA.

	N	Ka (M ^−1^)	ΔH (cal/mol)	ΔS (cal/mol/deg)
Z (70 nm)	0.132	32.0	−3.60 × 10^5^	−1.20 × 10^3^
Z (130 nm)	0.00318	32.2	−1.99 × 10^7^	−6.66 × 10^4^
Z (220 nm)	0.091	18.6	−2.54 × 10^5^	−884
